# Treatment Modality and Risk of Heart Failure in Patients With Long-Standing Graves’ Disease: A Nationwide Population-Based Cohort Study

**DOI:** 10.3389/fendo.2021.761782

**Published:** 2021-10-08

**Authors:** Eyun Song, Mina Kim, Sojeong Park, Min Jeong Park, Jung A. Kim, Eun Roh, Ji Hee Yu, Nam Hoon Kim, Ji A. Seo, Sin Gon Kim, Nan Hee Kim, Kyung Mook Choi, Sei Hyun Baik, Hye Jin Yoo

**Affiliations:** ^1^ Division of Endocrinology and Metabolism, Department of Internal Medicine, Korea University College of Medicine and School of Medicine, Seoul, South Korea; ^2^ Data Science Team, Hanmi Pharm. Co., Ltd, Seoul, South Korea

**Keywords:** Graves’ disease, hyperthyroidism, heart failure, anti-thyroid drug, radioiodine ablation

## Abstract

**Background:**

Optimal treatment for persistent Graves’ disease following 12–18 months of treatment with anti-thyroid drugs (ATDs) is unclear. Given the increased risk of cardiovascular morbidity and mortality with hyperthyroidism, assessing the risk of cardiovascular events associated with different treatment modalities after the conventional ATD course would be valuable in determining the appropriate next-line therapy.

**Methods:**

This retrospective cohort study included data from the Korean National Health Insurance database of 16,882 patients with newly diagnosed hyperthyroidism who received primary ATD treatment for 24 months. Patients were categorized based on the treatment they received after receiving ATD for 24 months: continued ATD for at least 12 more months (ATD group), radioiodine ablation (RIA) with remission (RIA group 1), and RIA without remission (RIA group 2). The incidence and risk of heart failure (HF), the leading cause of cardiovascular mortality in hyperthyroidism, were compared between patients and age-and sex-matched controls.

**Results:**

There were 16,516 (97.8%) patients in the ATD group, 230 (1.4%) in RIA group 1, and 136 (0.8%) in RIA group 2. Compared to that of controls, a significant difference in the cumulative incidence of HF was observed according to second-line treatment modality after adjusting for covariates; the risk was highest in patients in RIA group 2, with a hazard ratio (HR) of 2.54 (95% confidence interval (CI) 1.60–4.03), followed by those in the ATD group, with an HR of 1.23 (95% CI 1.20–1.36). Patients in RIA group 1 were not at an increased risk of HF compared to their matched controls (HR 0.77; 95% CI 0.38–1.54). When patients in the ATD group were further classified by the duration of ATD treatment at one-year intervals, the risk of HF was higher in patients with longer ATD use (p for linear trend < 0.001).

**Conclusions:**

In patients with long-standing hyperthyroidism treated with conventional duration of ATD therapy, the risk of HF was attenuated by RIA with remission of hyperthyroidism and increased as ATD was required for longer duration. To reduce the risk of HF, resolution of hyperthyroidism with RIA should be considered in patients with long-standing Graves’ disease.

## Introduction

Hyperthyroidism is prevalent in 0.2–1.3% of the population in iodine-replete areas, with Graves’ disease accounting for more than 80% of cases (1, 2). Patients with hyperthyroidism, especially those with uncontrolled or prolonged hyperthyroidism, are at an increased risk of cardiovascular morbidity and mortality (3–5). Three treatment modalities exist for Graves’ disease: anti-thyroid drug (ATD) therapy, radioactive iodine ablation (RIA), and thyroidectomy. Current guidelines do not prioritize any one treatment method among these three options as a first-line therapy as all methods have unique advantages and disadvantages (6–8). Nevertheless, ATD is currently used as an initial therapy for Graves’ disease in North America, Europe, and Asia (9–11).

A major drawback of ATD is the low rate of remission (12). With the recommended 12–18 month course of ATD therapy, a remission rate of approximately 30%–40% can be achieved (13). This is problematic considering the importance of prompt resolution of hyperthyroidism to reduce cardiovascular morbidity and mortality in these patients (5). It is recommended that patients with persistently high thyrotropin receptor antibody (TRAb) levels at 12–18 months can continue ATD treatment for an additional 12–18 months or opt for definitive therapy, e.g. RIA or thyroidectomy. However, treatment guidelines do not indicate a clear designation for the subsequent second-line therapy in this situation (6, 7). Previous studies have reported favorable outcomes of long-term (>24 months) treatment with ATDs with low rates of drug-related adverse events in patients with persistent or relapsed hyperthyroidism following a conventional course of ATD treatment (12, 14–17). However, the major outcome variable for measuring the efficacy of long-term ATD therapy in these studies was mostly the remission rate of hyperthyroidism, and the efficacy was not compared with other treatment modalities such as RIA or thyroidectomy. Given the increased risk of cardiovascular events in patients with hyperthyroidism and the improvement in survival associated with effective control of hyperthyroidism, it would be valuable to assess the risk of cardiovascular events in patients with long-standing hyperthyroidism according to different treatment methods following a conventional ATD course. To the best of our knowledge, no previous studies have assessed the direct impact of the type of second-line therapy on cardiovascular risk in the clinical setting of long-standing hyperthyroid patients. Thus, this study aimed to compare the risk of HF, the leading cause of cardiovascular mortality in hyperthyroidism (18–20), in patients who continued ATD after conventional duration of ATD therapy versus those who underwent subsequent RIA.

## Materials and Methods

### Data Source

Data for this nationwide retrospective cohort study were obtained from the Korean National Health Insurance Service (NHIS) database, which contains healthcare data of almost 98% of the national population from 2002 to 2019 (21). The NHIS database is managed by the Korean government and contains anonymized personal healthcare data, including health examination details, demographics, primary and secondary diagnoses based on International Classification of Diseases Tenth Revision (ICD-10) codes, inpatient and outpatient claims data, and medical treatments. As a part of this program, all participants enrolled in the NHIS are recommended to undergo general health examinations at least biannually. The results of general health examinations and questionnaires concerning lifestyle and behavior are registered in the National Health Screening Database. This study protocol was approved by the Institutional Review Board of Korea University (IRB file number 2020GR0206). A waiver for the requirement of informed consent was granted by the IRB because the NHIS provided researchers with anonymous, de-identified data using only anonymous identification numbers.

### Study Design and Population

**Figure 1:**
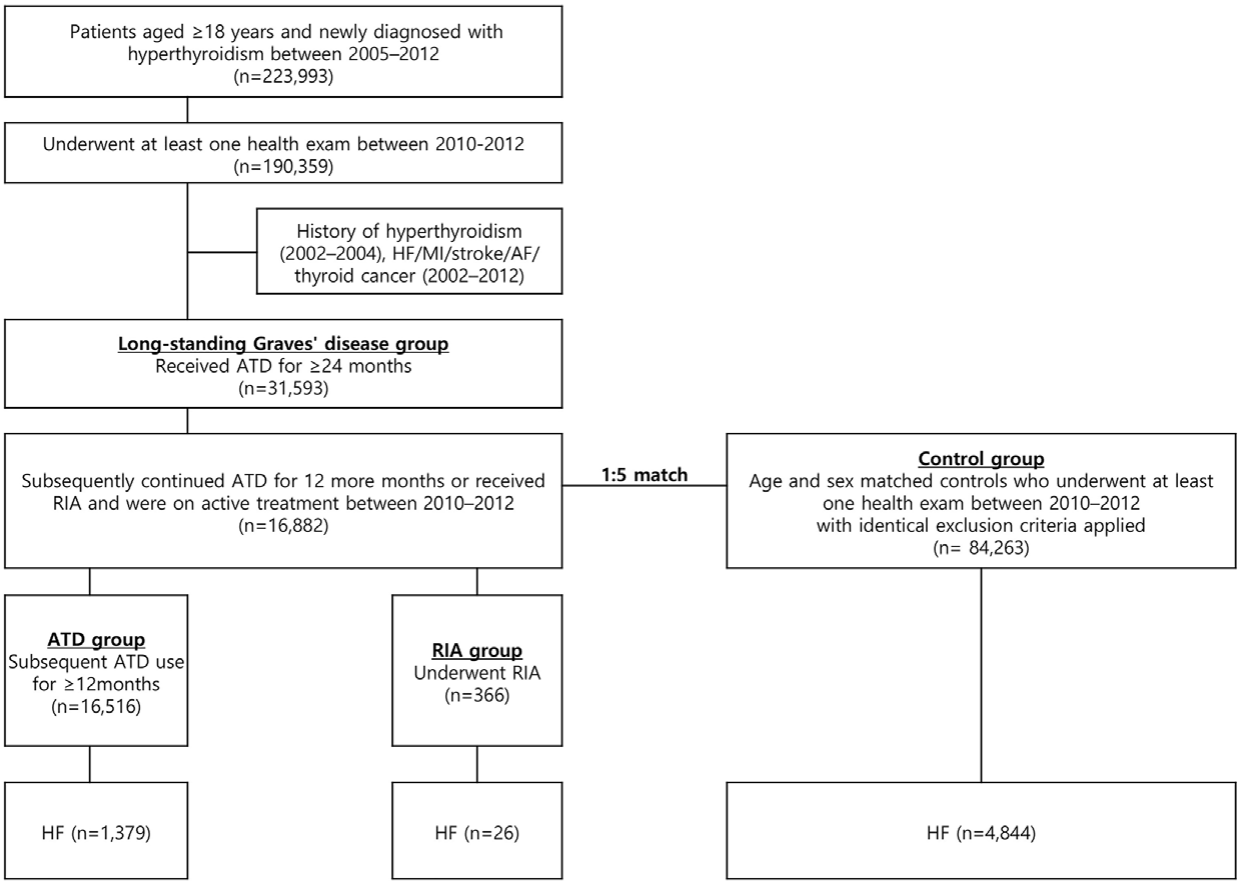
Flowchart of the study. ATD, anti-thyroid drug; AF, atrial fibrillation; HF, heart failure; MI, myocardial infarction; RIA, radioactive iodine ablation.

Data from the NHIS database from 2002 to 2019 was analyzed. We extracted records for patients diagnosed with hyperthyroidism using ICD-10 codes E05.0, E05.8, and E05.9. Patients diagnosed with hyperthyroidism between January 2005 and December 2012 who started ATD within 90 days of diagnosis were included in our analysis (**Figure 1**). A three-year washout period (2002–2004) was applied to select newly diagnosed cases of hyperthyroidism. Patients with long-standing Graves’ disease were defined as those who continued ATD treatment for ≥24 months. As 12–18 months is conventionally regarded as the upper limit of the duration of ATD treatment for Graves’ disease and current guidelines state that ATDs may not be helpful beyond 18–24 months (13, 22, 23), we regarded those who required ATD for more than 24 months as likely cases of long-standing Graves’ disease. Following ATD use for 24 months, patients who continued ATD treatment for at least 12 additional months without RIA treatment were placed in the ATD group. Those who underwent RIA at any point after the initial 24 month ATD treatment period were placed in the RIA group. Among these patients, those who were on ATDs or received RIA between 2010 and 2012 (index year) and underwent at least one National Health Screening Examination during this period were included. Individuals who were younger than 18 years old, had a history of HF (ICD-10 I50) or other major cardiovascular events, including myocardial infarction (MI, ICD-10 I21 or I22), ischemic or hemorrhagic stroke (ICD-10 I60–I63), or atrial fibrillation (ICD-10 I48), had a history of thyroid cancer (ICD-10 C73), or died before the index year were excluded. Controls without hyperthyroidism were selected through 5:1 direct matching to cases based on age group and sex. The same exclusion criteria were applied to controls before matching, and only those who underwent a National Health Screening Examination between 2010 and 2012 were included.

### Subgrouping

Patients in the RIA group were further separated into two groups according to the remission of hyperthyroidism: RIA group 1 included patients who achieved remission of hyperthyroidism within 1 year after ablation, and RIA group 2 included patients who did not achieve remission within 1 year after ablation. Remission of hyperthyroidism after ablation was defined as the discontinuation of ATD therapy. We determined the timeframe of 1 year after ablation as an appropriate window to assess the success or failure of the procedure, as previously described (5). Patients in the ATD group were further categorized based on the duration of ATD treatment as follows: ATD for 3 years (minimum duration for this study), 3–4 years, 4–5 years, and >5 years. We intended to classify patients in the RIA group in the same manner; however, this group was small, and there were too few patients who developed HF within each category of ATD duration to be considered a reliable classification for the analysis.

### Outcomes and Follow-up

The primary outcome was hospitalization for HF. This was captured when hospitalization with ICD-10 code for HF (I50) was the primary diagnosis. Follow-up for all cases was carried out until the records indicated development of outcome, death, or December 2019, whichever occurred first.

### Covariates

Baseline covariates such as body mass index (BMI), blood pressure (BP), cholesterol levels, fasting glucose levels, smoking status, alcohol consumption, and exercise status were obtained from the National Health Screening Examination data collected between 2010 and 2012 for both cases and controls. Heavy alcohol consumption was defined as an average daily alcohol intake of ≥30 g, and regular exercise was defined as moderate to strenuous physical activity ≥3 times/week. If participants underwent health screening examinations more than once during this period, the most recent data was used. Comorbidities were defined as the presence of at least two of the following diagnoses by ICD-10 codes within 2 years before the latest health screening examination: E10–14 for diabetes mellitus (DM), I10–13 or I15 for hypertension, and E78 for dyslipidemia.

### Statistical Analysis

Data were analyzed using SAS (version 9.4; SAS Institute Inc., Cary, NC, USA). Continuous variables were presented as median with interquartile ranges or mean with standard deviation, analyzed by a Mann–Whitney U test or Student’s t-test, respectively. Categorical variables were presented as counts with percentages and compared using a Pearson’s χ^2^ test. The curves for cumulative incidence of HF were constructed using the Kaplan–Meier method, and a log-rank test was used to compare the incidence between the groups. Cox proportional hazard models were used to calculate the hazard ratio (HR) and 95% confidence interval (CI) for HF multivariable adjustment: model 1 adjusted for age and sex; model 2 adjusted for age, sex, BMI, smoking status, alcohol consumption, regular exercise, DM, hypertension, and dyslipidemia; and model 3 additionally adjusted for systolic BP, low-density lipoprotein level, and fasting glucose level.

## Results

### Baseline Characteristics

This cohort included 16,882 patients with long-standing hyperthyroidism and 84,263 controls (**Figure 1**). Among 16,882 patients, 16,516 (97.8%) patients were in the ATD group and 366 (2.2%) were in the RIA group (230 in RIA group 1 and 136 in RIA group 2). **Table 1** summarizes the participants’ baseline characteristics. The median age was 51 years in both the hyperthyroidism and control groups, and women accounted for 65.9% of the cohort. Patients with long-standing hyperthyroidism had a higher prevalence of DM, hypertension, and dyslipidemia than did controls at baseline (all *p* < 0.001). The mean duration of ATD treatment was 4 ( ± 1.3) years with propylthiouracil being the most common ATD (71.6%), followed by methimazole (67.8%).

**Table 1:** Baseline characteristics.

Characteristics	Control (n = 84,263)	Hyperthyroidism (n = 16,882)	*p*-value	ATD group (n = 16,516)	RIA group (n = 366)	*p*-value
Age (years) [median (IQR)]	51 (42–60)	51 (42–60)	0.9835	52 (42–60)	45 (36–54)	<0.001
<30	3,955 (4.7%)	791 (4.7%)	0.9996	750 (4.5%)	41 (11.2%)	<0.0001
30–39	11,180 (13.3%)	2,265 (13.4%)		2,192 (13.3%)	73 (19.9%)	
40–49	21,725 (25.8%)	4,345 (25.7%)		4,236 (25.6%)	109 (29.8%)	
50–59	26,230 (31.1%)	5,246 (31.1%)		5,153 (31.2%)	93 (25.4%)	
60–69	14,960 (17.8%)	2,992 (17.7%)		2,953 (17.9%)	39 (10.7%)	
≥70	6,213 (7.4%)	1,243 (7.4%)		1,232 (7.4%)	11 (3.0%)	
Sex						
Male [n (%)]	28,775 (34.1%)	5,755 (34.1%)	0.8818	5,597 (33.9%)	158 (43.2%)	0.0002
Female [n (%)]	55,488 (65.9%)	11,127 (65.9%)		10,919 (66.1%)	208 (56.8%)	
Smoking			<0.0001			0.0011
Never smoker [n (%)]	61,710 (73.2%)	11,648 (69%)		11,427 (69.2%)	221 (60.4%)	
Ex-smoker [n (%)]	13,053 (15.5%)	3,070 (18.2%)		2,981 (18%)	89 (24.3%)	
Current smoker [n (%)]	9,500 (11.3%)	2,164 (12.8%)		2,108 (12.8%)	56 (15.3%)	
Heavy alcohol consumption [n (%)]	11,118 (13.2%)	1,591 (9.4%)	<0.0001	1,560 (9.4%)	31 (8.5%)	0.5275
Regular exercise [n (%)]	22,170 (26.3%)	4,110 (24.3%)	<0.0001	4,013 (24.3%)	97 (26.5%)	0.3309
Body mass index (kg/m^2^) [mean (SD)]	23.6 ( ± 3.2)	23.7 ( ± 3.2)	<0.0001	23.7 ( ± 3.2)	23.7 ( ± 3.3)	0.7011
Systolic BP (mmHg) [mean (SD)]	121.1 ( ± 15.1)	122.6 ( ± 14.7)	<0.0001	122.6 ( ± 14.7)	120.9 ( ± 13.7)	0.0233
Diastolic BP (mmHg) [mean (SD)]	75.4 ( ± 10.1)	75.5 ( ± 9.6)	0.0853	75.6 ( ± 9.6)	74.6 ( ± 9.2)	0.0513
Total cholesterol (mg/dL) [mean (SD)]	198.2 ( ± 36.7)	191.4 ( ± 36.9)	<0.0001	191.5 ( ± 36.8)	187.3 ( ± 40.8)	0.0521
HDL-C (mg/dL) [mean (SD)]	56.7 ( ± 17.4)	56.5 ( ± 22.5)	0.2785	56.5 ( ± 22.7)	57.6 ( ± 15.2)	0.1839
LDL-C (mg/dL) [mean (SD)]	117.3 ( ± 37.8)	112.3 ( ± 40.7)	<0.0001	112.4 ( ± 40.8)	108.4 ( ± 35.6)	0.0369
Fasting glucose (mg/dL) [mean (SD)]	97.1 ( ± 21.8)	98.6 ( ± 23.4)	<0.0001	98.7 ( ± 23.6)	95.1 ( ± 15.4)	<0.0001
Comorbidities						
Diabetes mellitus [n (%)]	6,861 (8.1%)	3,238 (19.2%)	<0.0001	3,193 (19.3%)	45 (12.3%)	0.0007
Hypertension [n (%)]	16,251 (19.3%)	5,541 (32.8%)	<0.0001	5,420 (32.8%)	121 (33.1%)	0.9218
Dyslipidemia [n (%)]	12,951 (15.4%)	5,661 (33.5%)	<0.0001	5,579 (33.8%)	82 (22.4%)	<0.0001
Duration of ATD treatment (years) [mean (SD)]*	–	3.99 ( ± 1.3)		4.01 ( ± 1.3)	3.02 ( ± 1.2)	<0.0001
ATDs**						
Methimazole [n (%)]	–	11,452 (67.8%)		11,111 (67.3%)	341 (93.2%)	<0.0001
Carbimazole [n (%)]	–	1,397 (8.3%)		1,334 (8.1%)	63 (17.2%)	
PTU [n (%)]	–	12,081 (71.6%)		11,845 (71.7%)	236 (64.5%)	

*For the RIA group, the duration was calculated from the initiation of ATD therapy to the date of ablation.

**The frequency of drug use was calculated in duplicate when more than one type of drug were used in one patient.

ATD, anti-thyroid drug; BP, blood pressure; HDL, high-density lipoprotein; IQR, interquartile range; LDL, low-density lipoprotein; PTU, propylthiouracil; RIA, radioactive iodine ablation; SD, standard deviation.

Patients in the ATD group were older than those in the RIA group (median 52 years vs. 45 years, *p* < 0.001) and had a higher proportion of female patients (66.1% vs. 56.8%, *p* < 0.001). The mean duration of ATD treatment was 4 ( ± 1.3) years in the ATD group and 3 ( ± 1.2) years in the RIA group (*p* < 0.001).

### Risk of Heart Failure According to Treatment Modality

**Figure 2:**
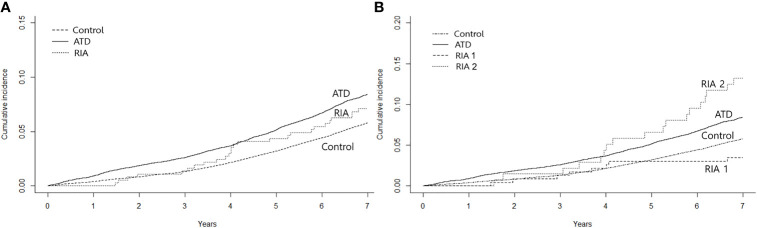
Kaplan–Meier curves for cumulative incidence of heart failure. **(A)** shows the incidence according to the treatment modality after 2 years of anti-thyroid drug therapy and **(B)** shows the incidence by taking into account whether remission was achieved after ablation. ATD, group that continued to receive anti-thyroid drug; RIA, group that underwent radioactive iodine ablation; RIA 1, group that underwent radioactive iodine ablation and achieved remission; RIA 2, group that underwent radioactive iodine ablation and did not achieve remission.

During the mean follow-up duration of 7.6 years, HF occurred in 1,379 (8.3%) patients in the ATD group, 26 (7.1%) in the RIA group, and 4,844 (5.7%) participants in the control group by data cutoff. **Figure 2A** shows the cumulative incidence of HF, and significant differences were observed among the three groups (log-rank *p* < 0.001). HRs for HF according to the Cox proportional hazards models are presented in **Table 2**. The ATD group had an HR of 1.28 (95% CI 1.2–1.36, *p* < 0.001), and the RIA group had an HR of 1.49 (95% CI 1.01–2.19, *p* = 0.044) compared to the control group after full adjustment (model 3). However, in between-group comparisons, HR for HF in the RIA group in reference to the ATD group showed no statistical significance (HR 1.11; 95% CI 0.75–1.64, *p* = 0.601).

**Table 2:** Hazard ratios and 95% confidence intervals for heart failure based on treatment modality.

	Control group	ATD group	RIA group
	(n = 84,263)	(n = 16,516)	(n = 366)
Unadjusted	1 (Ref.)	1.48 (1.4–1.57)	1.23 (0.84–1.81)
Model 1	1 (Ref.)	1.45 (1.36–1.54)	1.69 (1.15–2.49)
Model 2	1 (Ref.)	1.28 (1.21–1.37)	1.48 (1.00–2.17)
Model 3	1 (Ref.)	1.28 (1.2–1.36)	1.49 (1.01–2.19)
Unadjusted	—	1 (Ref.)	0.83 (0.57–1.23)
Model 1	—	1 (Ref.)	1.13 (0.77–1.67)
Model 2	—	1 (Ref.)	1.10 (0.75–1.63)
Model 3	—	1 (Ref.)	1.11 (0.75–1.64)

Model 1: adjusted for age, sex, and BMI.

Model 2: adjusted for age, sex, BMI, smoking status, alcohol consumption, regular exercise, diabetes mellitus, hypertension, and dyslipidemia;

Model 3: adjusted for age, sex, BMI, smoking status, alcohol consumption, regular exercise, diabetes mellitus, hypertension, dyslipidemia, systolic blood pressure, low-density lipoprotein level, and fasting glucose level.

ATD, anti-thyroid drug; BMI, body mass index; Ref, reference; RIA, radioactive iodine ablation.

Further analyses were performed by subgrouping RIA patients according to successful and unsuccessful hyperthyroidism remission within 1 year following ablation (RIA group 1 and RIA group 2, respectively). As shown in **Figure 2B**, a significant difference in the cumulative incidence of HF was observed among the four groups (log-rank *p* < 0.001). HR for HF after full adjustment was not significantly different in patients in RIA group 1 compared to the control group (HR 0.77, 95% CI 0.38–1.54) (**Table 3**). However, in RIA group 2, the HR was as high as 2.54% (95% CI 1.60–4.03, *p* < 0.001) compared to the controls and 1.91 (95% CI 1.20–3.05, *p* < 0.001) compared to the ATD group.

**Table 3:** Hazard ratios and 95% confidence intervals for heart failure among treatment groups; ATD *vs.* RIA with remission *vs.* RIA without remission.

	Control group	ATD group	RIA group 1	RIA group 2
	(n = 84,263)	(n = 16,516)	(n = 230)	(n = 136)
Unadjusted	1 (Ref.)	1.48 (1.4–1.57)	0.6 (0.3–1.19)	2.36 (1.49–3.75)
Model 1	1 (Ref.)	1.45 (1.36–1.54)	0.86 (0.43–1.73)	2.96 (1.86–4.69)
Model 2	1 (Ref.)	1.28 (1.21–1.37)	0.77 (0.38–1.53)	2.52 (1.59–4.01)
Model 3	1 (Ref.)	1.28 (1.2–1.36)	0.77 (0.39–1.54)	2.54 (1.6–4.03)
Unadjusted	—	1 (Ref.)	0.4 (0.2–0.81)	1.59 (1–2.53)
Model 1	—	1 (Ref.)	0.57 (0.29–1.15)	2 (1.26–3.19)
Model 2	—	1 (Ref.)	0.57 (0.28–1.14)	1.9 (1.19–3.03)
Model 3	—	1 (Ref.)	0.57 (0.29–1.14)	1.91 (1.2–3.05)

Model 1: adjusted for age, sex, and BMI.

Model 2: adjusted for age, sex, BMI, smoking status, alcohol consumption, regular exercise, diabetes mellitus, hypertension, and dyslipidemia.

Model 3: adjusted for age, sex, BMI, smoking status, alcohol consumption, regular exercise, diabetes mellitus, hypertension, dyslipidemia, systolic blood pressure, low-density lipoprotein level, and fasting glucose level.

ATD, anti-thyroid drug; BMI, body mass index; Ref, reference; RIA, radioactive iodine ablation.

### Association Between Duration of Anti-Thyroid Drug Use and Risk for Heart Failure

**Figure 3:**
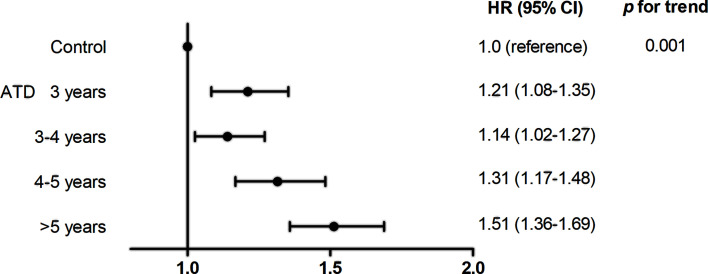
Association between duration of anti-thyroid drug use and risk for heart failure. ATD, anti-thyroid drug; HR, hazard ratio; CI, confidence interval.

In the ATD group, risk of HF was assessed according to the duration of ATD treatment (**Figure 3**). With the control group as a reference, patients treated with ATD for 3 years had an HR of 1.21 (95% CI 1.08–1.35), for 3–4 years of 1.14 (95% CI 1.02–1.27), for 4–5 years of 1.31 (95% CI 1.17–1.48), and for >5 years of 1.51 (95% CI 1.36–1.69) (*p* for trend = 0.001).

## Discussion

This study evaluated the risk of HF in patients with long-standing Graves’ disease according to secondary treatment modality following an initial 24-month course of ATD therapy. Compared to their age-and sex-matched controls, patients with long-standing Graves’ disease had a 28%–49% increased risk of HF after adjusting for various confounding factors. Notably, however, patients who achieved remission from hyperthyroidism following RIA within 1 year were not at increased risk for HF, while those who did not achieve remission had a 2.54-fold increased risk. Furthermore, in patients who received ATDs continuously after 24 months of the initial ATD course, the longer ATDs were used, the higher was the risk of HF. Our results show that in patients with long-standing Graves’ disease, early and definitive treatment with RIA should be considered to reduce the risk of HF.

Hyperthyroidism is associated with an increased risk of cardiovascular morbidity and mortality (3). Whether this risk is affected by different therapies for hyperthyroidism has not been fully elucidated. A previous study by Boelaert et al. compared the all-cause mortality rate between ATD therapy and RIA in patients with hyperthyroidism and observed a survival benefit with post-RIA hypothyroidism relative to ATD therapy or RIA not resulting in hypothyroidism (24). However, this study did not specifically investigate cardiovascular risks, and the number of cases was underpowered to show survival gain in the Graves’ disease subset. Ryödi et al. examined the risk of cardiovascular diseases (CVDs) in patients treated with RIA compared to those who underwent thyroidectomy and showed a higher CVD risk in the former but did not include patients treated with ATDs (25). A recent large-scale study (n = 4,189) of patients with Graves’ disease compared the overall survival and occurrence of major adverse cardiovascular events (MACEs) 1 year after diagnosis of Graves’ disease in patients treated with ATDs versus RIA (5). In reference to the control group, the risks of all-cause mortality and MACEs were highest in patients with unresolved hyperthyroidism after RIA by 1 year, followed by patients treated with ATDs. In patients with resolved hyperthyroidism after RIA within 1 year, the survival risk was not elevated, and the risk for MACEs was the lowest. Our study is distinguishable from previous studies in that we assessed the risk of HF according to *second-line* treatment modality. We selected a unique cohort of patients already treated with first-line ATDs for 24 months, which may represent the real-world clinical practice in which ATD therapy is used as the primary mode of treatment by 54%–97% of clinicians worldwide (8, 10). Our results showed that the risk of HF can be ameliorated by RIA when remission is achieved in patients with chronic hyperthyroidism and that the risk of HF is increased in those requiring a long duration of ATD treatment.

Current guidelines recommend 12–18 months of ATD therapy if used as primary treatment for hyperthyroidism, but there is no general agreement as to which treatment is best for chronic hyperthyroidism after this period (6–8). A recent prospective, randomized trial showed that long-term methimazole for 60–120 months lowered the relapse rate 3-fold at 48 months compared to conventional 18–24 month courses of treatment in patients with Graves’ disease (23). A multicenter cohort study of 908 patients with Graves’ disease in Korea also reported that longer use of ATDs was associated with a lower relapse rate (*p* = 0.003). The authors of both studies suggest that long-term (>24 months) ATD therapy is safe and effective in terms of reducing relapse rates. However, as hyperthyroidism is closely associated with cardiovascular events, achieving remission should not be the sole factor when considering treatment modalities. Clinicians must also consider increased risk of cardiovascular events when determining optimal second-line therapies for patients with long-standing hyperthyroidism. Our study focused on this point and observed significant differences in the risk of HF according to treatment method.

Notably, the risk of HF increased as ATDs were used for a longer duration in patients in the ATD group (**Figure 3**). As thyroid function test (TFT) data were not available in our dataset, we could not accurately assess whether biochemical remission was achieved in these patients. However, because ATDs were withdrawn at a certain time point and no further RIA was performed, we can assume that remission was likely to be achieved. If this is the case, using ATDs longer than 24 months can increase the remission rate as reported (12, 14–17, 23) but may not be beneficial to control HF events. In the aforementioned study comparing all-cause mortality in patients treated with ATD and RIA, patients with persistently low thyroid stimulating hormone levels after 1 year of treatment had a 55% increased risk for death, regardless of treatment method (5). This highlights the importance of rapid and effective control of hyperthyroidism to improve survival. In this respect, priority should be given to early resolution of hyperthyroidism in patients already treated with ATDs for the upper limit duration of conventional therapy, as in our study. Further studies with a comprehensive investigation of the remission rate and risk of cardiovascular events with long-term ATD treatment in a single cohort may assist in determining the optimal time to step forward to definitive treatment.

This study had several limitations. First, data on TFT and TRAb were unavailable; thus, our study lacks information on the severity of hyperthyroidism at diagnosis, whether RIA was administered because of heavily uncontrolled hyperthyroidism, and whether biochemical remission was achieved after the treatment. Second, the diagnosis was based on ICD-10 codes, and accurate information about the etiology of hyperthyroidism in these patients is limited. However, Graves’ disease accounts for 82.7% of hyperthyroidism cases in Korea and toxic adenomas for less than 1% (8). Patients who used ATDs for more than 24 months with a diagnosis of hyperthyroidism in Korea would be expected to have Graves’ disease. Third, due to the retrospective design, we could not eliminate the possible bias from clinician preference and patient compliance to ATDs. Fourth, we did not include patients who underwent thyroidectomy for the treatment of hyperthyroidism, due to very small number of these patients. Lastly, we did not evaluate the risk of other CVDs. Growing evidence supports that hyperthyroidism accelerates atherosclerosis (26) and thus increases the risk of MI and stroke (5, 27–29), albeit controversial (18, 30, 31). Further studies evaluating the risk of MI or stroke may provide additional valuable information. Despite these limitations, strengths of this study are the large sample size and a unique cohort of patients with long-standing Graves’ disease. To the best of our knowledge, this is the first study to evaluate the risk of HF according to second-line therapies.

In conclusion, in patients with long-standing hyperthyroidism who underwent a conventional ATD course, the risk of HF differed according to the secondary treatment modality. Risk of HF was lowest in patients with controlled hyperthyroidism after RIA, increased as ATD was required for a prolonged duration, and was highest in patients with uncontrolled hyperthyroidism after RIA. In addition to the goal of achieving remission, is it important to consider the risk of comorbidities when determining the best therapies for long-standing hyperthyroid patients is necessary.

## Data Availability Statement

The data analyzed in this study is subject to the following licenses/restrictions: containing information could compromise the privacy of research participants. Requests to access these datasets should be directed to HY, deisy21@naver.com.

## Ethics Statement

The studies involving human participants were reviewed and approved by Institutional Review Board of Korea University. Written informed consent for participation was not required for this study in accordance with the national legislation and the institutional requirements.

## Author Contributions

Conceptualization: ES and HY. Data curation: ES, MP, JK, ER, SB, and HY. Formal Analysis: ES, MK, and SP. Investigation: ES, MP, JK, ER, JY, NamK, HY, JS, SK, NanK, SB, and HY. Methodology: ES, MK, SP, and HY. Project administration: HY. Resources: ES, MP, JK, ER, JY, NamK, KC, JS, SK, NanK, SB, and HY. Supervision: HY. Visualization: ES, MK, and SP. Writing – original draft: ES and HY. Writing – review & editing: ES, MP, JK, ER, JY, NamK, JS, SK, NanK, KC, SB, and HY. All authors contributed to the article and approved the submitted version.

## Funding

This work was supported by National Research Foundation of Korea (NRF) funded by the Ministry of Education of Korea (2021R1A2C2008792).

## Conflict of Interest

Authors MK and SP were employed by company Hanmi Pharm. Co., Ltd.

The remaining authors declare that the research was conducted in the absence of any commercial or financial relationships that could be construed as a potential conflict of interest.

## Publisher’s Note

All claims expressed in this article are solely those of the authors and do not necessarily represent those of their affiliated organizations, or those of the publisher, the editors and the reviewers. Any product that may be evaluated in this article, or claim that may be made by its manufacturer, is not guaranteed or endorsed by the publisher.
